# Pustular dermatitis due to *Pseudomonas oryzihabitans* in makeup

**DOI:** 10.1097/JW9.0000000000000024

**Published:** 2022-05-19

**Authors:** Roberto Russo, Andrea Muracchioli, Paola Canepa, Aurora Parodi, Anna Maria Spagnolo

**Affiliations:** Department of Health Science (DISSAL), University of Genoa, Genoa, Italy, Unit of Dermatology, Ospedale Policlinico San Martino, Genoa, Italy; Department of Health Science (DISSAL), University of Genoa, Genoa, Italy

**Keywords:** *Pseudomonas oryzihabitans*, pustular dermatitis

**What is known about this subject in regard to women and their families?**

- Skin infections caused by *Pseudomonas oryzihabitans* are extremely rare.
- They usually happen in immunocompromised patients, for example, AIDS patients, people who consume alcoholics or patients with other conditions depleting lymphocyte count, following trauma, surgery, or animal bites.
- Nonpuerperal mastitis caused by *P. oryzihabitans* has been described in young, healthy women.

**What is new from this article as messages for women and their families?**

- Even in immunocompetent patients, skin infections by *P. oryzihabitans* may be favored by microtraumas such as bristles of a colonized makeup brush. Microtraumas play an important role in colonization of skin by bacteria, also due to the use of masks during the pandemic, and/or application of aggressive products to treat skin inflammation caused by the masks.
- Makeup brushes should be considered as potential sources of skin infection due to their possibility of colonization and microtraumas they may cause; therefore, they should be removed when a skin infection develops, and they should not be shared with other people. Also, they are not to be considered eternal, and should be replaced periodically.

## Dear Editors,

**Fig. 1.**
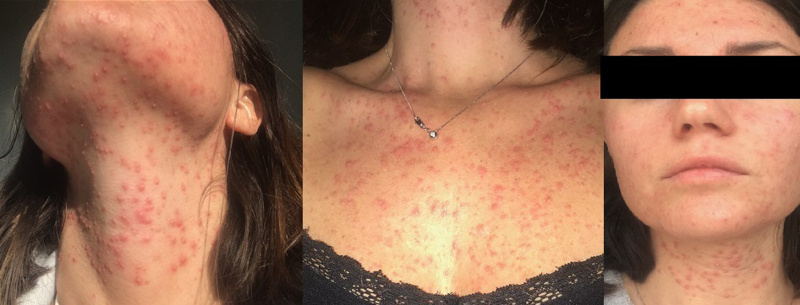
Pustular eruption involving forehead, face, anterior neck, and chest.

A 30-year-old female patient presented with an extremely pruritic rash involving face, neck, and chest. Her medical history was unremarkable. She denied taking medications or consuming alcohol. At clinical examination, the eruption was composed of pustules and involved forehead, face, anterior neck and chest, with sharp borders and sparing of scalp, shoulders, and back (Fig. 1). Physical examination was otherwise unremarkable. Laboratory tests, including blood count and CD4+ count, were within normal ranges. HIV tests were negative. Gram staining and swab culture, performed on MacConkey agar, of pus from a lesion revealed the presence of nonlactose fermenting GRAM-negative bacteria. The isolate was identified as *Pseudomonas oryzihabitans* by the VITEK 2-automated microbiology system (BIOMÉRIEUX). Given the distribution of the eruption, culture tests were also performed from the makeup products of the patient, and a foundation brush was found to be colonized by *P*. *oryzihabitans*. A 6-week topical therapy with amikacin was prescribed according to the susceptibility test, leading to dramatic improvement.

*P.* (formerly *Flavimonas*) *oryzihabitans* is a yellow-pigmented, Gram-negative, oxidase-negative, nonfermenting bacillus, which has been isolated from damp environments, such as rice paddies and sink drains. Diverse epidemiological studies have found *P*. *oryzihabitans* in the hospital environment. Occasionally, it may cause catheter-associated infections.^1^

*P*. *oryzihabitans* causes skin infections very rarely. Principally, it has been found in surgical wound infections or mastitis, usually favored by local predisposing factors, or by immunosuppression (eg, AIDS, diabetes, and drugs).^2^ Pustular rashes caused by *P*. *oryzihabitans* were only reported, together with sepsis, in a 1-year-old child from a rural area of Ghana.^3^ We are reporting a unique case of a diffuse pustular dermatitis due to *P*. *oryzihabitans* in an immunocompetent adult patient. She did not have any condition impairing her immune system, nor wounds, bites, or traumas on the affected areas. Maybe, the only condition predisposing infection could have been a pre-existing “maskne,” an acne-like eruption arising on the skin under the masks widely used due to the COVID-19 pandemic, or rosacea. The pustular dermatitis also involved forehead, neck, and chest. This made us suspect makeup products as the possible source of colonization, also considering a previous report of a synthetic bath sponge as the source of catheter contamination by *P*. *oryzihabitans* and subsequent bacteremia in an AIDS patient.^1^ Therefore, we tested all the makeup products and equipment, including sponges, used by the patient, which were all noncolonized except the foundation brush. Of course, it is possible that the brush might have been contaminated due to contact with the skin already infected. However, we could not find an alternative explanation for the colonization of skin. In fact, the patient was a school teacher and during the pandemic was working from home, so there was no professional exposition. Also, other people living in the same house had no manifestation, and all the other products tested (including bath sponges) were not contaminated. The colonized foundation brush is probably the source of skin infection, as the pustules were localized on the areas where the makeup was applied. Microtrauma caused by the bristles of the brush could have allowed *P*. *oryzihabitans* to penetrate the skin. Removing the brush, together with the topical antibiotic treatment, resulted in prompt resolution of the rash.

## Conflicts of interest

None.

## Funding

None.

## Study approval

N/A

## Patient Consent

Informed, written consent was received from the patient and confirmed to the journal pre-publication, stating that the patient gave consent for her photos and case history to be published.
